# MDM2 Promotes Invasion and Metastasis in Invasive Ductal Breast Carcinoma by Inducing Matrix Metalloproteinase-9

**DOI:** 10.1371/journal.pone.0078794

**Published:** 2013-11-13

**Authors:** Xiaofeng Chen, Jinrong Qiu, Dapeng Yang, Jianlei Lu, Caiyun Yan, Xiaoming Zha, Yongmei Yin

**Affiliations:** 1 Department of Oncology, The First Affiliated Hospital of Nanjing Medical University, Nanjing, China; 2 Department of Developmental Genetics, Nanjing Medical University, Nanjing, China; 3 Department of General Surgery, The First Affiliated Hospital of Nanjing Medical University, Nanjing, China; Wayne State University School of Medicine, United States of America

## Abstract

The molecular mechanisms that underpin invasive ductal breast cancer (IDC) invasion and metastasis are incompletely understood. The oncogene, mouse double minute 2 (*MDM2*), has been implicated in the pathogenesis of numerous cancers, where it stimulates the expression of matrix metalloproteinase 9 (MMP9), an important enzyme in the breakdown of the extracellular matrix. However, its role in breast cancer remains poorly understood. This study assessed the clinical significance of MDM2 expression in IDC and used in vitro expression assays to determine the molecular roles of MDM2. Immunohistochemical staining for MMP9 and MDM2 was performed using archived tumor blocks from 321 women who underwent surgical resection for IDC at the First Affiliated Hospital of Nanjing Medical University, China between January 2002 and December 2003. MCF-7 and MDA-MD-231 cell lines were transfected with siRNA targeted against MDM2, or MDM2 was overexpressed using transiently expressed vectors. The invasion, cell migration and proteolytic capabilities of cells that over- or underexpressed MDM2 was then assessed and compared against control cells, in addition to the consequent effects on MMP9 expression using RT-PCR. In vivo, 54.9% and 49.6% of samples were positive for MMP9 and MDM2 expression, respectively, and their expression was significantly correlated (r^2^ = 0.171, P = 0.012). Moreover, MDM2 expression was markedly correlated with disease-free survival (HR 2.56, 95% CI 1.02–6.40, P = 0.038). In vitro, MDM2 overexpression significantly enhanced cell invasion, migration and proteolysis compared with control cells, and the converse effects were observed after MDM2-siRNA treatment. MDM2 overexpression induced MMP9 expression in a dose-dependent manner. Taken together, these results suggest that high levels of MDM2 are associated with a poorer prognosis in IDC. This might result from increased tumor invasiveness due to enhanced MMP9 expression causing increased extracellular matrix breakdown.

## Introduction

Breast cancer is the most frequent cancer that occurs in women worldwide, and is the second leading cause of female cancer deaths at all ages [1], mostly as a consequence of metastasis [2], [3]. It has been estimated that 25%–40% of patients with breast cancer will develop metastatic disease, which is generally incurable [4]. Breast cancer principally metastasizes to the regional lymph nodes, bone, liver, lungs and brain [5]. The 5-year survival rate for patients with non-metastatic breast cancer drops to 20% for those with metastatic disease [6]. The process of metastasis is highly complex, consisting of a number of distinct steps that include the invasion of surrounding stromal tissue, intravasation, evasion of programmed cell death, arrest within the vasculature at a distant site, extravasation and the establishment and growth of tumor tissue at secondary sites [7]–[10]. The underlying molecular mechanisms of metastasis at each of these stages in breast cancer are relatively poorly understood, and the identification of genes that regulate breast cancer invasion and metastasis will help in the design of strategies that can prevent and treat late-stage breast cancer.

*MDM2* (mouse double minute 2; the human gene is also known as *HDM2*) is an oncogene which is expressed at high levels in numerous human cancers, including those of the breast, lung, colon, pancreas, and hematological malignancies lymphomas and leukemias [11]. Moreover, numerous studies have shown that MDM2 overexpression is associated with tumors that have a higher degree of invasiveness, later stages, greater metastatic potentiality and resistance to chemotherapeutic agents and radiation [12]. MDM2 possess E3 ubiquitin ligase activity, which promotes the proteasomal degradation of p53 by catalyzing its ubiquitination [13]; in turn, MDM2 is transactivated by p53 [14]. Although the mechanism by which MDM2 contributes to tumor initiation has been clarified with the identification of multiple interacting proteins [11], the precise roles of MDM2 in tumor invasion and metastasis remain unknown.

The MMPs are a family of structurally conserved, zinc-dependent endopeptidases, which are involved in proteolytic modeling of the extracellular matrix (ECM) [15]. MMP9, also known as 97 kDa type IV collagenase, plays important roles in tumor invasion [16]–[20]. Previous studies have shown that the expression of MMPs, including MMP9, can be upregulated by MDM2, although this has not been show in breast cancer to date.

The aim of this study was to ascertain the effects of MDM2 expression on the invasive potential of breast cancer cell lines in vitro, in addition to the effects of MMP9 expression. Furthermore, the levels of MDM2 and MMP9 expressed in clinical biopsy samples of invasive ductal breast carcinoma (IDC) were quantified and correlated. Our findings showed that MDM2 expression was correlated with MMP9 levels in clinical samples. MDM2 overexpression in vitro enhanced cell motility, invasion as well as MMP9 levels, and these effects were reversed by MDM2-siRNA blockade. These findings suggest an important role for MDM2 in breast cancer invasion and metastasis.

## Materials and Methods

### Patients and Materials

The study was approved by the review board and ethics committee of the First Affiliated Hospital of Nanjing Medical University, Nanjing, China, and was conducted in accordance with the Helsinki declaration on the use of human subjects in research. All patients gave their written, informed consent before participation. Overall, 321 female patients with IDC who underwent a surgical resection at the Department of Breast Surgery, the First Affiliated Hospital of Nanjing Medical University between January 1^st^, 2002 and December 31^st^, 2003, were recruited for this study. Nine patients (2.8%, data not shown) with incomplete clinicopathological data were excluded. Overall, 70 patients (21.8%, data not shown) were not followed-up. The mean duration of follow-up was 45 months (range, 2–84 months). Analysis of the present report focused on the remaining 242 patients (median age 51.0 years, range 22–79 years) who were followed-up. Of these, 60 patients received no adjuvant therapy, while 182 patients underwent systemic adjuvant therapies, including standard adjuvant chemotherapy, radiotherapy or hormonal treatment. The clinicopathological features of all patients are listed in Table 1.

**Table 1:** Correlation between the expression of MMP9 and MDM2 with clinicopathological factors in tissue samples of IDC.

Variable	MMP9			*P*-value	MDM2			*P*-value
	−		+		−		+	
Age				0.894				0.340
≤50 yr	46	65			44	63		
>50 yr	50	68			53	57		
Tumor size				0.688				**0.028**
≤2 cm	54	71			43	72		
>2 cm	42	62			54	48		
Lymph node involvement				0.076				0.268
Negative	64	72			53	75		
Positive	32	61			44	45		
ER status				0.879				0.878
Negative	72	98			71	89		
Positive	24	35			26	31		
PR status				1.000				1.000
Negative	75	104			76	94		
Positive	21	29			21	26		
HER2 status				0.864				0.288
Negative	79	108			77	102		
Positive	17	25			20	18		

ER: estrogen receptor; PR: progesterone receptor; HER2: human epidermal growth factor receptor 2.

For all of the selected cases, we recovered the original hematoxylin and eosin (H&E)-stained slides and new histological slides were made from the corresponding formalin fixed paraffin-embedded (FFPE) tissue blocks. The disease was staged according to the TNM Classification of Malignant Tumors published by the International Union against Cancer (6th edition, 2006). All laboratory investigators were completely blinded to the clinical outcomes.

### Immunohistochemical Analysis of MMP9 and MDM2

After deparaffinazation and rehydration of the sections from FFPE samples, antigen retrieval was performed in a pressure cooker. Endogenous peroxidase activity was blocked with 0.5% hydrogen peroxide for 10 min. Antigens were recovered by heating the slides in an autoclave sterilizer for 2 min in 0.01 mol/l Tris-HCl at pH 6.0. The sections were incubated overnight at 4°C with a primary anti-MMP9 antibody (1∶150; Cell Signaling Technology, USA) or an anti-MDM2 antibody (1∶100; Santa Cruz Biotechnology, CA, USA). The sections were then incubated with the secondary antibody (Dako Co., Glostrup, Denmark) for 30 min and then visualized with 3, 3′-diaminobenzidine (DAB). Negative controls were prepared using phosphate-buffered saline (PBS) instead of the respective primary antibody, while sections from tissues previously recognized as positive for the selected antibodies were used as positive controls.

Immunohistochemical assays were evaluated using a semi-quantitative scale by two independent investigators (ZH Zhang & GX Song). The percentage of stained cells was recorded and each sample was classified according to a specific expression pattern for each antibody and the number of positive cells [21]. Staining of the cell membrane, cytoplasm or both in more than 10% of cells was considered as a sample that was positive for MMP9, while >1% nuclear and/or cytoplasmic staining was considered positive for MDM2 [22].

### Ethics Statement

The study was approved by the Review Board of the First Affiliated Hospital of Nanjing Medical University and the Review Board of Nanjing Medical University.

### Wound Healing Assay

MCF-7 cells (ATCC, American Type Culture Collection) were seeded in 6-well tissue culture plates to achieve 90%–95% confluency before transfection on the following day. Transfection was performed with MDM2 expression plasmids or siRNA specific to MDM2 (Accession number: NM_002392) and scrambled controls using Lipofectamine 2000 (Invitrogen, California, America). Twenty-four hours after transfection, scratches were created in a monolayer of confluent cells using a 200-µl pipette tip. The cells were then rinsed with fresh medium to remove any free-floating cells and debris. Wound healing was observed at different time points within the scrape line, and representative scrape lines were photographed. Measurement of wound area was done using the Adobe Photoshop software. Wound closure was quantified as the mean ± standard deviation of three independent experiments. The control wound closure was set at 100%, and the MDM2 treatments are represented as the percent of the control.

### Cell Invasion Assay

MDA-MB-231 cells (ATCC, American Type Culture Collection) were seeded on 6-well tissue culture plates to achieve 90%–95% confluency on the day prior to transfection. Transfection was performed with MDM2 expression plasmids or siRNA specific to MDM2 and a corresponding scrambled control using Lipofectamine 2000. MDA-MB-231 cells were trypsinized 24 h after transfection and seeded at a density of 3×10^5^ cells onto extracellular matrix (ECM) gel coated inserts (8-µm pore size) in 24-well tissue culture plates. The cells were incubated in DMEM medium supplemented with 2% serum. After 24 h incubation, the cells on the upper side of the inserts were removed with a cotton swab. The cells that migrated to the lower side of the inserts were stained with 1% crystal violet and counted using light microscopy.

### Western Blot Assay

Cells were lysed with RIPA buffer (50 mM Tris-HCl, pH 7.5, 150 mM NaCl, 1% NP-40, 0.5% sodium deoxycholate and 0.1% SDS) containing protease inhibitors (Roche). Total protein (20 µg) was boiled for 5 min in 1×loading buffer, chilled on ice, and then separated on 8% SDS-polyacrylamide gels. Following transfer onto PVDF membranes (Millipore, Billerica, America), nonspecific protein interactions were blocked by incubation in 5% nonfat dry milk in TST-buffer (50 mM Tris-HCl, 150 mM NaCl, 0.05% Tween-20 pH 7.6) at room temperature (RT) for 1 h. Membranes were then incubated overnight at 4°C with primary antibody in fresh blocking buffer. Unbound antibody was removed by three 10 min washes in TST buffer. Membranes were then incubated with horseradish peroxide-conjugated individual secondary antibody for 1 h at RT, followed by three 10 min washes with TST buffer. The blot was developed with ECL reagent (Pierce, Rockford, America). Prestained markers were used as molecular weight standards.

### Luciferase Assay

MCF-7 cells were plated on 12-well tissue culture plates to achieve 90%–95% confluency before transfection on the following day. Transfection was performed with MMP9-Luc reporter plasmids along with MDM2 expression plasmids or control plasmids using Lipofectamine 2000. The β-galactosidase vector was used as an internal control for transfection efficiency. Cells were washed with PBS 24 h after transfection and lysed using 1 × lysis buffer; 20 µl of cell extract was then assayed for luciferase activity using a Luciferase Assay Kit (Promega, Wisconsin state, America) according to the manufacturer’s instructions. β-galactosidase activity was measured to normalize the luciferase activity. This experiment was repeated three times and carried out in triplicate.

### Zymography Assay

MDA-MB-231 cells were seeded in 6-well tissue culture plates and allowed to adhere in the presence of serum. Twenty-four hours after transfection with MDM2 expression plasmids or siRNA against MDM2 or a scrambled control, cells were incubated with serum-free media. After 24 h incubation, supernatants were collected and centrifuged to remove cellular debris. Concentrated samples with equal amounts of proteins were mixed with SDS sample buffer without reducing agent and subjected to 8% SDS-PAGE containing 0.1% gelatin A (Sigma, St Louis, Mo, USA). After electrophoresis, gels were washed three times in 2.5% Triton X-100 for 1 h at RT to remove the SDS, and then incubated for 24–48 h at 37°C in buffer containing 5 mM CaCl_2_ and 1 mM ZnCl_2_. Thereafter, gels were fixed and stained with 0.25% Coomassie blue R-250 for 4 h and then destained in 45% methanol and 10% acetic acid. Proteolytic activity was displayed as clear bands (zones of gelatin degradation) against the blue background of the stained gelatin. The molecular weights were estimated with reference to prestained SDS-PAGE markers.

### RNA Isolation and RT-PCR Analysis

Total RNA was isolated with Trizol reagent (Invitrogen, Carlsbad, USA) according to the manufacturer’s protocol. cDNAs were synthesized from total RNA (1 µg) using random primers with the ReverTra Ace-α-TM First Strand cDNA Synthesis Kit (Toyobo Co. Ltd., Japan). RT-PCR was performed using the MMP9 primer set (forward, 5′-CGT CTT CCC CTT CAC TTT CC-3′; reverse, 5′-CAC AGT AGT GGC CGT AGA AG-3′) with the following PCR conditions: denaturation at 94°C for 30 s, annealing at 53°C for 30 s, and extension at 72°C for 30 s. After 32 cycles, an additional extension at 72°C for 10 min was performed. β-actin was amplified simultaneously as an internal control.

### Statistical Analysis

Statistical analysis was performed with SPSS for Windows (SPSS Inc., Chicago, IL, USA). Two-tailed χ^2^ tests were used to compare the expression levels of MMP9 and MDM2 in biopsy samples with clinicopathological factors. Correlations between the expression levels of these proteins were assessed by calculating the Spearman correlation coefficient. Events of disease-free survival (DFS) were defined as locoregional relapse, metastatic relapse, contralateral breast cancer and death from any cause. Univariate and multivariate analysis were performed using Cox’s regression model for survival to determine the hazard ratios (HR) and 95% confidence intervals (CI). Two-tailed P-values of <0.05 were considered statistically significant.

For the in vitro studies, results are expressed as the mean ± SEM or SD. Data were analyzed using one-way analysis of variance between groups (ANOVA) with the least significant difference test. P-values <0.05 were considered to be statistically significant.

## Results

### MMP9 and MDM2 Expression in Tissue Samples of IDC

**Figure 1:**
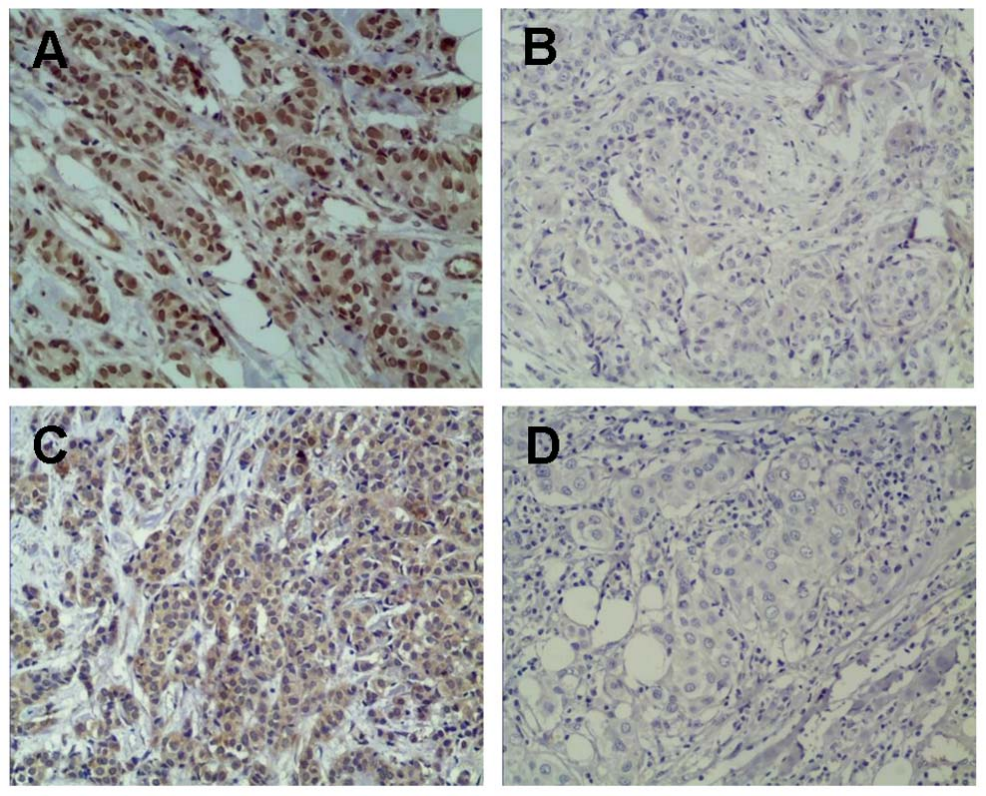
Representative immunohistochemistry images. MDM2 expression was demonstrated by brown-staining in the cell membrane or cytoplasm (A), while MMP-9 expression was shown as brown-staining in the nucleus or cytoplasm (C). The negative staining patterns are presented to enable a comparison (B, MDM2 negative, and D, MMP-9 negative).

Positive expression of MMP9 was demonstrated by brown-staining in the cell membrane or cytoplasm, while positive MDM2 expression was shown as brown-staining in the nucleus or cytoplasm (Figure 1). In tissue samples of IDC, the positive rates of MMP9 and MDM2 expression were 54.9% and 49.6%, respectively. We noted a significant correlation between the expression of MMP9 and MDM2 (r^2^ = 0.171, P = 0.012).

As shown in Table 1, the expression of MMP9 and MDM2 were not significantly associated with age, lymph node involvement, ER status, PR status or HER2 status (P>0.05). However, MDM2 expression was significantly associated with tumor size (P = 0.028).

**Figure 2:**
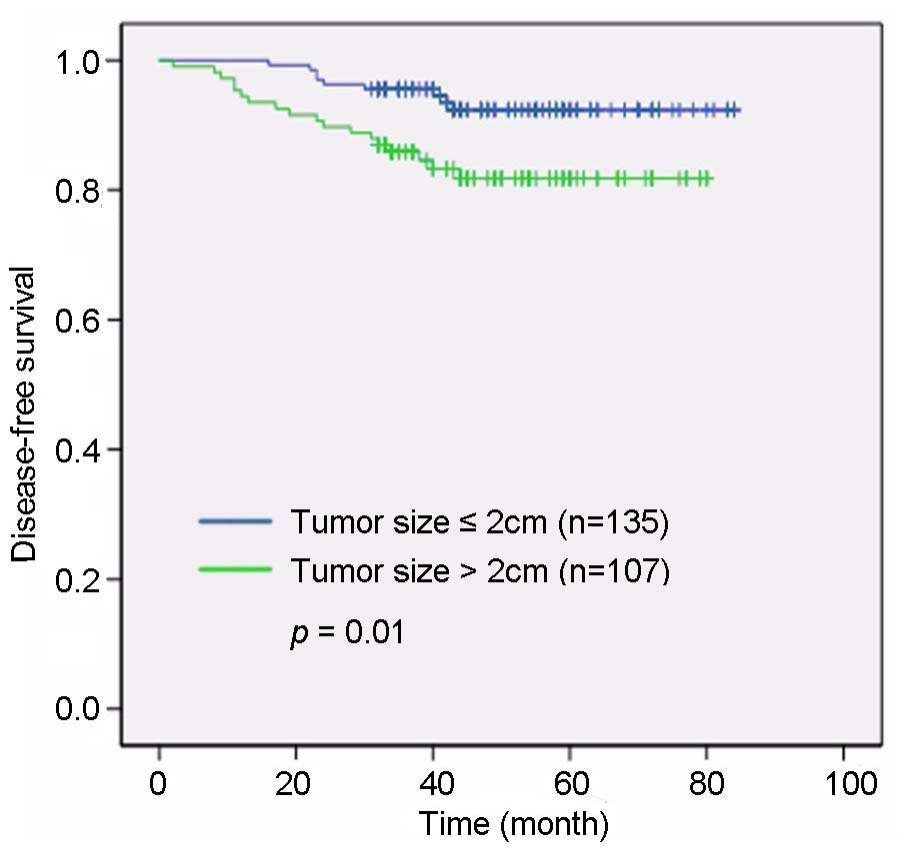
Univariate analysis showed a significant association between tumor size and disease-free survival. Tumor sizes >2 cm were correlated with a shorter disease-free survival duration than found in patients with tumor sizes ≤2 cm (P = 0.01).

**Figure 3:**
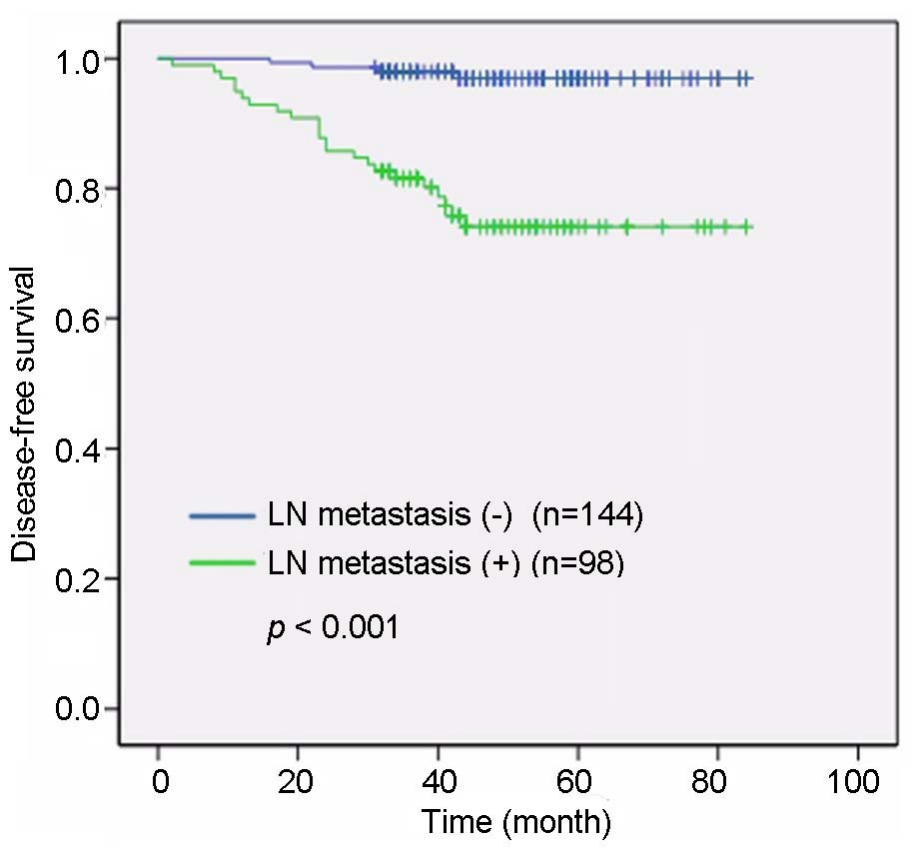
A significant association was determined between lymph node involvement and disease-free survival. A univariate analysis showed that patients with lymph node involvement had shorter disease-free survival durations than patients without lymphatic metastases (P<0.01).

**Figure 4:**
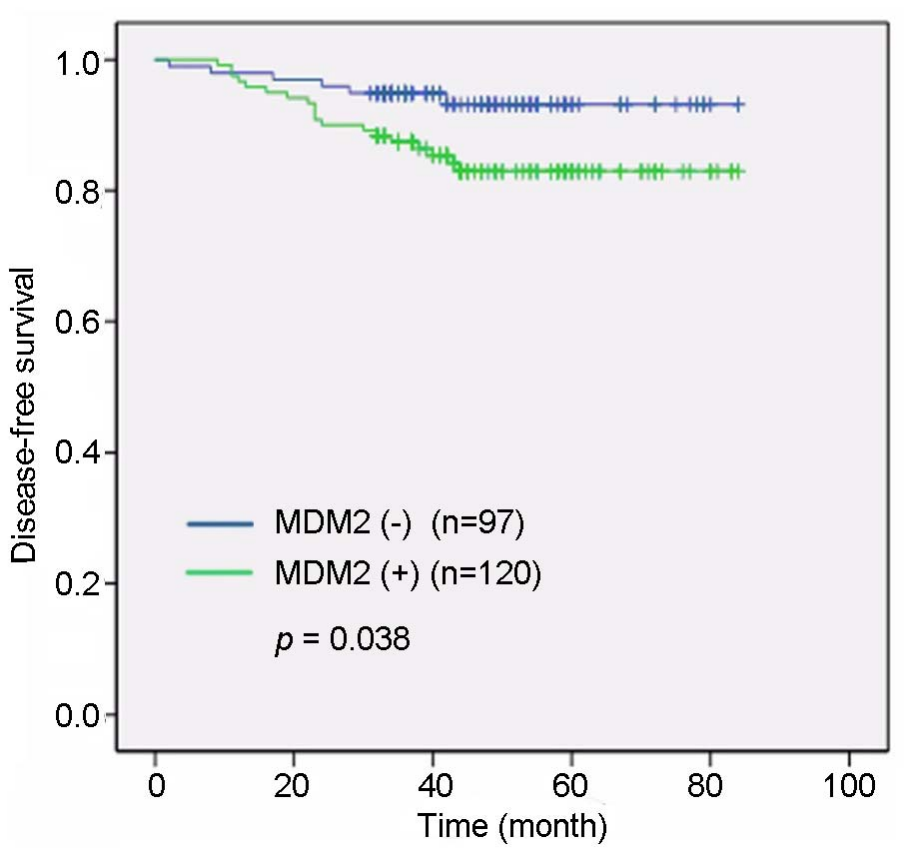
MDM2 expression levels were significantly correlated with disease-free survival. Univariate analysis showed that patients with MDM2-positive expression had shorter disease-free survival times than patients with tumors that were MDM2-negative (P = 0.038).

Univariate analysis showed a significant association between tumor size and DFS rates (P = 0.01, Figure 2). Lymph node involvement was also correlated with the DFS rate (P<0.001, Figure 3). The expression of MDM2 (HR 2.56, 95% CI 1.02–6.40, P = 0.038, Figure 4) showed a marked effect on DFS rates (Table 2). Multivariate analysis using the stepwise backward elimination method revealed that lymph node involvement (HR 10.892, 95% CI 3.39–35.00, P<0.001) and MDM2 expression (HR 4.86, 95% CI 1.63–14.49, P = 0.005) were independent prognostic factors for survival in IDC (Table 3).

**Table 2:** Univariate analysis using Cox proportional risk modeling in patients with IDC.

Variable	Patientsn = 242 (%)	Relapsen = 27	*P*-value	HR(95% CI)
Age			0.545	
≤50	118 (48.8)	15		1.00
>50	124 (51.2)	12		0.79 (0.37–1.69)
Tumor size			**0.01**	
≤2 cm	135 (55.8)	9		1.00
>2 cm	107 (44.2)	18		**2.73 (1.23–6.08)**
Lymph nodeinvolvement			**<0.001**	
Negative	144 (59.5)	4		1.00
Positive	98 (40.5)	23		**9.57 (3.31–27.68)**
ER status			0.100	
Negative	179 (74.0)	24		1.00
Positive	63 (26.0)	3		0.38 (0.11–1.26)
PR status			0.333	
Negative	188 (77.7)	23		1.00
Positive	54 (22.3)	4		0.60 (0.21–1.72)
HER2 status			0.865	
Negative	200 (82.6)	22		1.00
Positive	42 (17.4)	5		1.09 (0.41–2.87)
MMP9 expression			0.234	
Negative	96 (39.7)	8		1.00
Positive	133 (54.9)	18		1.65 (0.72–3.79)
Unknown	13 (5.4)	1		
MDM2 expression			**0.038**	
Negative	97 (40.1)	6		1.00
Positive	120 (49.6)	19		**2.56 (1.02–6.40)**
Unknown	25 (10.3)	2		
Breast cancer subtypes				
Luminal A	75 (31.0)	5		1.00
Luminal B	6 (2.5)	1	0.447	2.31 (0.27–19.81)
Basal-like	125 (51.7)	17	0.216	1.37 (0.83–2.26)
ERBB2+	36 (14.9)	4	0.479	1.17 (0.76–1.82)

HR: hazard ratio; 95%CI: 95% confidence interval; ER: estrogen receptor; PR: progesterone receptor; HER2: human epidermal growth factor receptor 2.

**Table 3:** Multivariate analysis using the stepwise backward elimination method in patients with IDC.

Variable	Partial regressioncoefficient	Wald value	HR	95%CI	*P*-value
Age	−0.054	0.016	0.947	0.407–2.205	0.900
Tumor size	0.540	1.339	1.716	0.688–4.282	0.247
Lymph node involvement	2.388	16.081	10.892	3.390–34.996	**<0.001**
ER Status	−0.979	1.926	0.376	0.094–1.497	0.165
PR Status	−0.300	0.189	0.741	0.192–2.861	0.664
HER2 Status	0.673	1.385	1.960	0.639–6.014	0.239
MMP9 expression	−0.422	0.747	0.656	0.252–1.707	0.387
MDM2 expression	1.582	8.065	4.864	1.633–14.490	**0.005**

HR: hazard ratio; 95%CI: 95% confidence interval; ER: estrogen receptor; PR: progesterone receptor; HER2: human epidermal growth factor receptor 2.

### MDM2 Promotes MCF-7 Cell Migration

**Figure 5:**
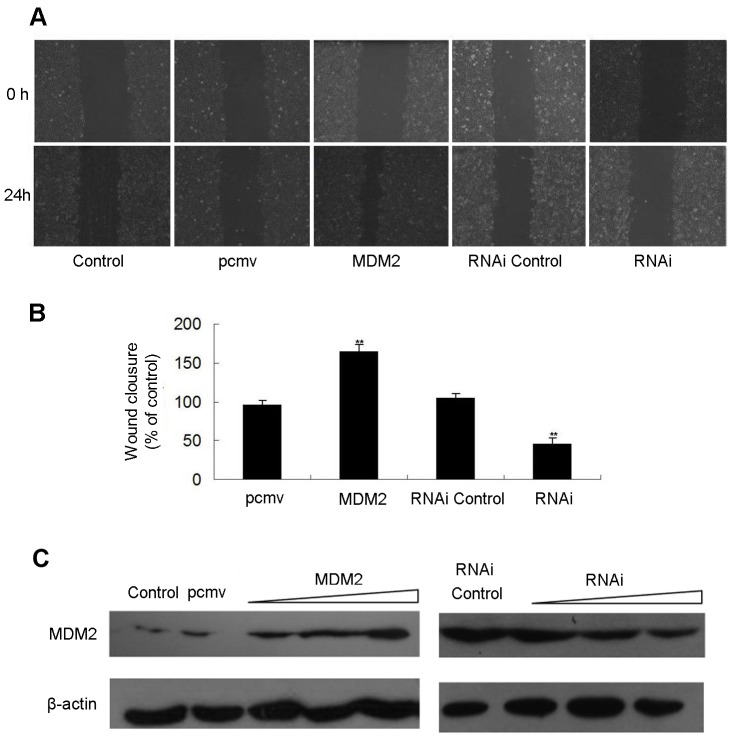
MDM2 promotes the migration of MCF-7 cells. MCF-7 cells were transfected with pcmv-MDM2 expression plasmids and pcmv vectors or siRNAs against MDM2 and non-specific siRNA (controls); after 24 h, the cells were scraped with a sterile pipette tip to create a wound. (A) Wound closure was observed by phase-contrast microscopy and photographed at 0 and 24 h. (B) The wound area was measured by the Adobe Photoshop software. Wound closure was quantified as the mean ± standard deviation of three independent experiments. The control wound closure was set at 100%, and the MDM2 treatments are represented as the percent of the control. (C) The levels of MDM2 protein were detected using Western blot analysis in MCF-7 cells that over- or under-expressed MDM2 (plasmid or siRNA transfection) and control cells. β-actin levels served as internal control.

The effects of MDM2 transfection on MCF-7 cell migration were assessed by the wound healing assay. As shown in Figure 5A, photomicrographs taken at 24 h after the scratch was created showed increased wound closure in MCF-7 cells transfected with pcmv-MDM2 plasmids compared with empty vector-transfected control cultures. Conversely, delayed wound closure was observed in cells transfected with MDM2 siRNA compared with cells transfected with the non-specific control siRNA. The quantitation of the wound closure over time revealed a significant stimulatory effect of MDM2 on MCF-7 motility (Figure 5B). Results of Western blot analysis showed that the expression of MDM2 in MCF-7 cells was increased or decreased after pcmv-MDM2 plasmids or MDM2 siRNA transfection (Figure 5C).

### MDM2 Promotes Invasion of MDA-MB-231 Cells

**Figure 6:**
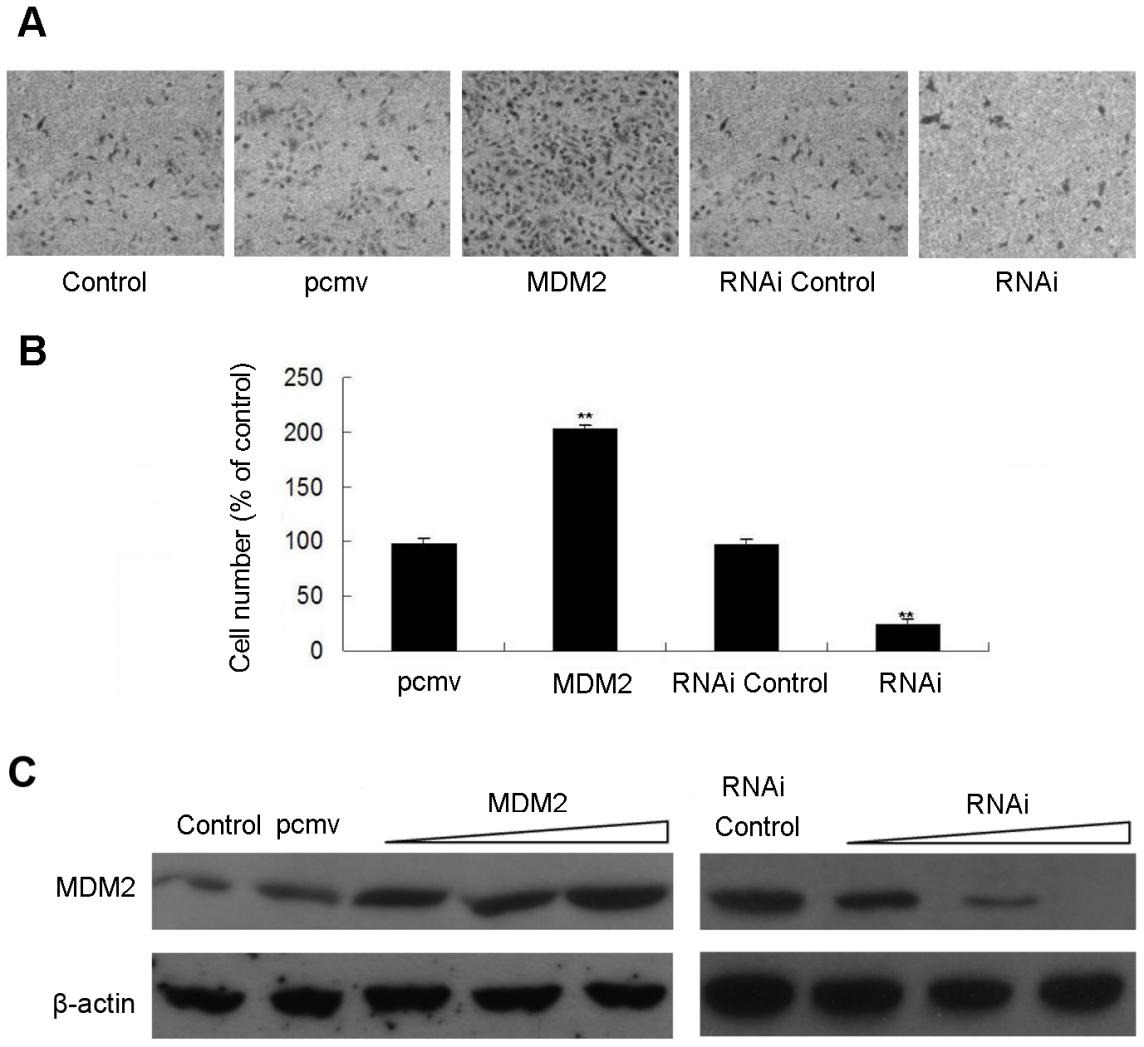
MDM2 promotes the invasion of MDA-MB-231 cells. MDA-MB-231 cells were transfected with pcmv-MDM2 expression plasmids and pcmv vectors, siRNAs against MDM2 or non-specific siRNA (controls). After 48 h, the cells were plated onto a Matrigel to assay cell invasion. Cells were allowed to migrate for 20 h at 37°C. Cells on the upper side of the inserts were removed with a cotton swab. Cells that migrated to the lower side of the filters were stained with 1% crystal violet and counted under light microscopy. (A) Digital pictures of cell invasiveness. Stained areas represent the numbers of invasive cells, and all images are representative of three independent experiments; **(**B) Number of invasive cells compared to the control (expressed as the percentage of the control). (C) The levels of MDM2 protein were detected using Western blot analysis in MDA-MB-231 cells that over- or under-expressed MDM2 (plasmid or siRNA transfection) and control cells. β-actin levels served as internal control.

To examine the role of MDM2 in cell invasion, MDA-MB-231 cells, a highly metastatic breast cancer cell line, were transfected with pcmv-MDM2 expression plasmids or siRNA for MDM2 and their corresponding controls for 24 h. A transwell assay was then performed. MDM2 overexpression significantly enhanced MDA-MB-231 cell invasion compared with the control cells (Figure 6A and 6B). Conversely, the invasion of MDA-MB-231 cells was inhibited in cells transfected with siRNA for MDM2 compared with cells transfected with non-specific siRNA (Figure 6A and 6B). The increased or decreased expression of MDM2 in MDA-MB 231 cells transfected with pcmv-MDM2 plasmids or MDM2 siRNA was shown in Figure 6C.

### MDM2 Upregulates MMP9 Promoter Activity, mRNA Level and Enzymatic Activity

**Figure 7:**
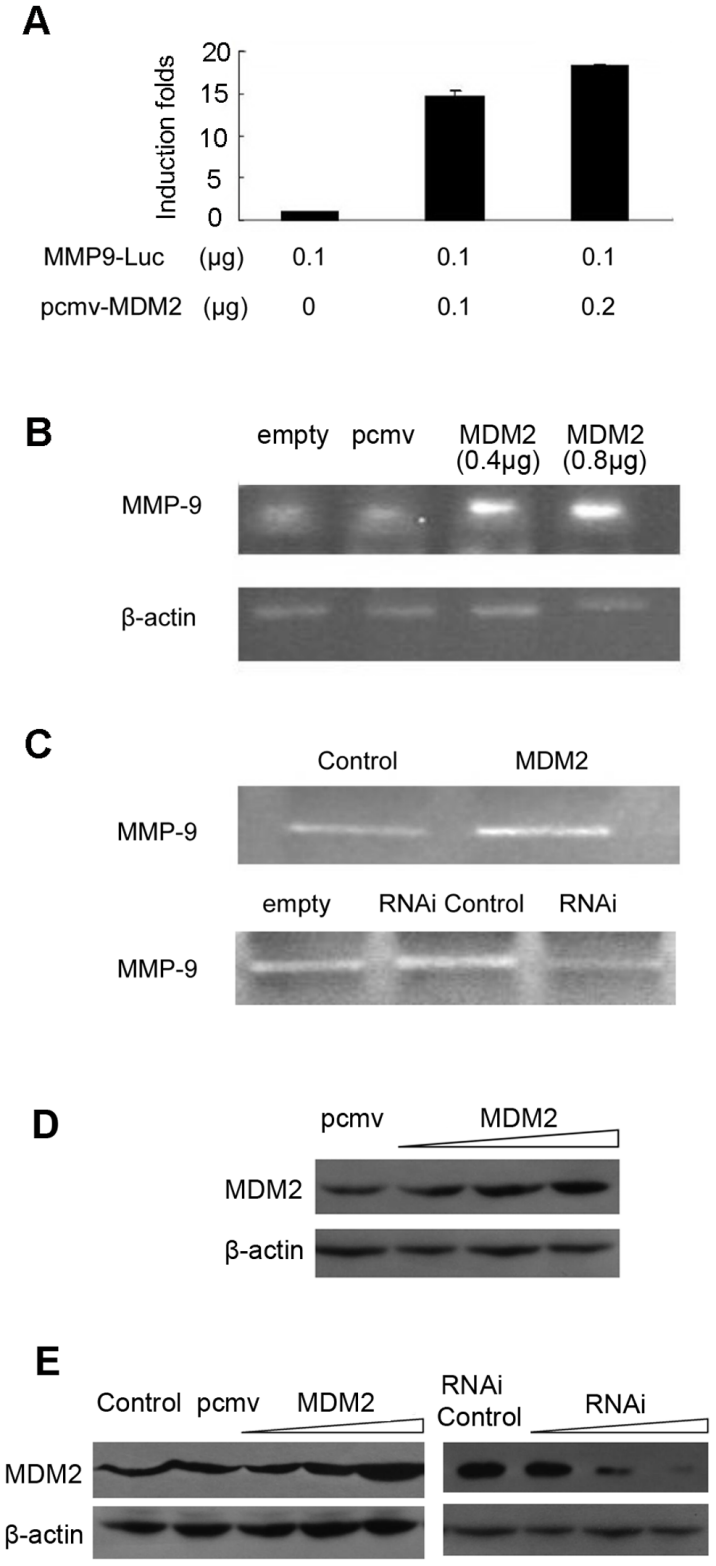
MDM2 upregulates MMP9 expression. (A) MCF-7 cells were transfected with MMP9-Luc reporter plasmids and pcmv-MDM2 expression plasmids for 24 h. Whole-cell lysates were utilized to detect luciferase activities, and β-galactosidase activities were used as an internal control for transfection efficiency. The results were obtained from three independent experiments. Data are expressed as the mean ± SD. (B) Total RNA was isolated from MCF-7 cells transfected with pcmv-MDM2 expression plasmids or pcmv vectors to assay MMP9 mRNA levels using RT-PCR, normalized against β-actin. Images are representative of three independent experiments. (C) MMP9 activity was detected using gelatin zymography in MDA-MB-231 cells that over- or under-expressed MDM2 (plasmid or siRNA transfection) and control cells. (D) The levels of MDM2 protein were detected using Western blot analysis in MDF-7 cells transfected with MDM2 expression plasmids and control vectors. β-actin levels served as internal control. (E) The levels of MDM2 protein were detected using Western blot analysis in MDA-MB-231 cells that over- or under-expressed MDM2 (plasmid or siRNA transfection) and control cells. β-actin levels served as internal control.

MCF-7 cells were transiently transfected with the *MMP9*-Luc reporter construct and increasing amounts of pcmv-MDM2 expression plasmids. As shown in Figure 7A, MMP9 promoter activity was markedly increased by MDM2. Furthermore, MDM2 enhanced MMP9 mRNA expression in a dose-dependent manner (Figure 7B). Using Gelatin Zymography, we found that MDM2 overexpression increased MMP9 enzymatic activity in MDA-MB-231 cells (Figure 7C). Conversely, MMP9 enzymatic activity was decreased as MDM2 expression was decreased by siRNA (Figure 7C). These results indicate that MDM2 promotes the invasion of MDA-MB- 231 cells by stimulating the expression and enzymatic activity of MMP9. The increased expression of MDM2 in MCF-7 cells transfected with pcmv-MDM2 plasmids was shown in Figure 7D. The increased or decreased expression of MDM2 in MDA-MB 231 cells transfected with pcmv-MDM2 plasmids or MDM2 siRNA was shown in Figure 7E.

## Discussion

The underlying molecular mechanisms that contribute to the invasion and metastasis of breast cancer cells are still being elucidated, and are likely to be highly polygenic. This current study investigated the role of MDM2, an oncogene first identified in mice, and showed that not only was its expression correlated with disease-free survival in clinical samples, but the overexpression of MDM2 enhanced the invasion and motility properties of two breast cancer cell lines in vitro. These effects were abolished by the transfection of cells lines with siRNA targeted against *MDM2*. Furthermore, expression levels of MMP9, an important enzyme for degrading the extracellular matrix, were correlated with high MDM2 levels in clinical IDC samples, and were enhanced by the increased expression of MDM2 in vitro in a dose-dependent manner.

*MDM2* was first cloned in a murine double-minute chromosome in 3T3DM cells, and was found to be tumorigenic in mice [23]. The MDM2 oncoprotein contains an N-terminal p53-binding domain [24], a nuclear localization signal (NLS), a nuclear export signal (NES), a central acidic domain, a C-terminal zinc-finger domain, and a RING finger domain that possesses E3 ligase activity [25]–[29]. The E3 ligase activity degrades p53, a critical tumor suppressor protein in breast tissue [30]. Antagonists of MDM2 activity have been shown to activate p53, inducing cell cycle arrest [31], and it is thought that the majority of the tumorigenic effects of MDM2 are a result of this interference.

However, recent research has indicated that MDM2 may induce the expression of other genes with importance in carcinogenesis. A microarray investigation into a cell model of pancreatic cancer indicated that MDM2 was upregulated along with 39 other metastasis-related genes, including 13 ECM-related genes of which MMP9 was one [32]. A mouse xenograft tumor model that overexpressed beta-arrestin, a known regulator of MDM2 [33], showed increased MMP9 activity with more aggressive tumors [34]. Furthermore, in vitro studies using pancreatic cell lines showed a direct link between MDM2 downregulation and the suppression of MMP9 expression [35]. In their latter study, Shi et al. also showed that overexpressed MDM2 had higher metastatic potential and were associated with higher MMP9 levels.

MMPs are essential in many aspects of tumor progression including remodeling of the ECM for tumor invasion [36]. Moreover, MMP9, a key member of the MMP family, plays a crucial role in the degradation of ECM and is upregulated in breast cancer as part of the extracellular matrix remodeling signature of this disease [37]. Thus, we examined the effect of MDM2 on MMP9 expression in vitro and assessed the correlation between the two proteins using immunohistochemical analysis of human breast cancer tissue.

MMP9 expression is regulated at both transcriptional and post-transcriptional levels [38]–[40], of which the former appears to be the major regulatory mechanism. The promoter region of MMP9 contains several transcription factor binding sites, including two AP-1 sites, an NF-κB site, an ETS site, and a Sp1 site. These elements are sufficient for the transcriptional activation of *MMP9* by various stimuli [41].

The pathogenesis of breast cancer is complex and polygenic. It is therefore not surprising that different genes to those studied in this present work have overlapping functions. Recent studies have identified two other oncogenes, KLF8 [42] and AIB1 [43] that upregulate the expression and activity of MMP9 and MMP2, another important ECM MMP involved in carcinogenesis. It would be important in future studies to perform array-based expression studies of metastatic IDC tissue to gain a fuller understanding of the oncogenes involved in this particular feature of breast cancer. More comprehensive cell line investigations could then be performed to assess the underlying mechanistic interactions between these oncogenes and their downstream effectors, such as the MMP protein family.

In conclusion, we have shown that increased expression of MDM2 in IDC tissue correlates with poorer disease-free survival outcomes, and with increased expression levels of MMP9. In vitro studies have confirmed that the overexpression of MDM2 confers a more aggressive phenotype to breast cancer cell lines, including higher levels of cell motility and invasion, in addition to inducing the expression and activity of MMP9. All of the effects occurred in a dose-dependent manner and were reversed by the siRNA-mediated blockade of MDM2 expression. We conclude that MDM2 plays an important role in the invasion and metastasis of breast carcinoma via the degradation of the surrounding extracellular matrix. Further studies will focus on delineating the wider downstream effects of this oncogene on the ability of breast cancers to metastasize.
